# *NKX2-5* Mutations in an Inbred Consanguineous Population: Genetic and Phenotypic Diversity

**DOI:** 10.1038/srep08848

**Published:** 2015-03-06

**Authors:** Ossama K. Abou Hassan, Akl C. Fahed, Manal Batrawi, Mariam Arabi, Marwan M. Refaat, Steven R. DePalma, J. G. Seidman, Christine E. Seidman, Fadi F. Bitar, Georges M. Nemer

**Affiliations:** 1 Department of Biochemistry and Molecular Genetics, American University of Beirut, Beirut, Lebanon; 2 Department of Genetics, Harvard Medical School, Boston, MA; 3 Department of Medicine, Massachusetts General Hospital, Boston, MA; 4 Department of Pediatrics and Adolescent Medicine, American University of Beirut, Beirut, Lebanon; 5 Department of Internal Medicine, American University of Beirut, Beirut, Lebanon; 6 Howard Hughes Medical Institute and Division of Cardiology, Brigham and Women's Hospital, Boston, MA

## Abstract

*NKX2-5* mutations are associated with different forms of congenital heart disease. Despite the knowledge gained from molecular and animal studies, genotype-phenotype correlations in humans are limited by the lack of large cohorts and the incomplete assessment of family members. We hypothesized that studying the role of *NKX2-5* in inbred populations with homogeneous genetic backgrounds and high consanguinity rates such as Lebanon could help closing this gap. We sequenced *NKX2-5* in 188 index CHD cases (25 with ASD). Five variants (three segregated in families) were detected in eleven families including the previously documented p.R25C variant, which was found in seven patients from different families, and in one healthy individual. In 3/5 familial dominant ASD cases, we identified an *NKX2-5* mutation. In addition to the heterogeneity of *NKX2-5* mutations, a diversity of phenotypes occurred within the families with predominant ASD and AV block. We did in fact identify a large prevalence of Sudden Cardiac Death (SCD) in families with truncating mutations, and two patients with coronary sinus disease. *NKX2-5* is thus responsible for dominant familial ASD even in consanguineous populations, and a wide genetic and phenotypic diversity is characteristic of *NKX2-5* mutations in the Lebanese population.

Atrial Septal Defect (ASD) is one of the most frequent congenital heart diseases (CHD) with an incidence of 1 in 1500 live births worldwide. This cardiac malformation which affects the atria early on during heart development accounts also for 30–40% of all CHD in adults^1^,^2^. Most neonates have minimal symptoms allowing thus further clinical complications to present later in life. In rare cases, however, and if the size of the septal defect is large, a significant amount of blood is shunted from the left side of the heart to the right leading to heart failure.

*NKX2-5* encodes an NK-2 homeodomain transcription factor known to control an evolutionary conserved cardiac regulatory network^3^,^4^. The *in vitro* characterization of *NKX*2-5 as a master regulator of the transcriptional activity of cardiac-specific genes like *NPPA*, and the early and broad expression of the gene in the heart was suggestive of a role in cardiogenesis. This was supported by the *NKX2-5-/-* knock-out mouse model which led to embryonic lethality at embryonic stage 10.5 (e10.5)^5^.

Additional studies unraveled the regulatory role of this gene in controlling cardiac cell specification, and chamber formation. Repeated identification of atrioventricular (AV) block in patients with *NKX2-5* mutations unraveled the role of the gene in the conduction system. High levels of *NKX*2-5 were found within the conduction system cells of developing human hearts^6^. In mice, loss of one copy of *NKX2-5* led to the scattering of the AV bundle and reduced HIS and AV node cellular density especially at the proximal posterior compartment^7^,^8^,^9^. A hypoplastic and disorganized Purkinje network was also observed in these mice^10^. Further analysis of the embryos showed a down-regulation and/or abnormal expression of different gap junction proteins (GJas) as the underlying cause of the abnormalities in impulse propagation, and subsequently the development of arrhythmias. Reduced levels of GJa1 were later shown to contribute to increased risk in ventricular arrhythmias and sudden death, while reduced GJa5 levels were linked to atrial electrical instability with increased risk of atrial fibrillation^11^. Furthermore, a lateral distribution of gap junctions at myocyte junction borders is thought to affect dissipation of the cardiac impulse within the ventricular sink, prolonging its propagation and heightening the risk of arrhythmogenesis through micro-reentry circuits^12^.

Fifty-eight different mutations in *NKX2-5* have been reported in the literature with a wide range of cardiac clinical presentations. The most commonly reported phenotypes are ASD and AV block in 68.4%, and 65.7% of the cases respectively^13^. Characterization of some of these mutations both *in vitro* and in mouse models has shed light on the functional domains of the protein as well as its partners. Roughly, one third of all reported missense mutations occur within the homeodomain region. Functional studies of these proteins showed reduced DNA binding and/or transcriptional activity without affecting translocation to the nucleus^14^,^15^,^16^,^17^,^18^. Missense mutations in the non-DNA binding domains affect protein dimerization and interaction with DNA^15^ resulting in reduction of transcriptional synergistic activity with its partners^16^,^17^,^18^.

Consanguinity has been shown to increase the risk of CHD across different populations^19^, including the Lebanese^20^, which has consanguinity rates that can range between 12.5 to 42% in rural areas^21^. Autosomal dominant genes can harbor benign or less deleterious heterozygous variants that can present in homozygous form in consanguineous populations. Also inbred populations often harbor founder mutations that contribute to a large fraction of a rare disease in the population. In this study we aimed to understand the structure and distribution of *NKX2-5* mutations in the CHD Lebanese population.

We sequenced *NKX2-5* in 188 sequential CHD patients who presented to a pediatric cardiology clinic as part of a targeted sequencing effort of a set of genes implicated in CHD. We identified three novel mutations segregating with disease in three autosomal dominant families with ASD. We revisit the functional role of *NKX2-5* mutations based on all reported variants in the gene and their location on the protein, and then undergo further phenotyping to delineate the development of arrhythmias and risk of sudden cardiac deaths in mutations leading to the truncation of the protein after the homeodomain region.

## Methods

### Study Subjects and Phenotyping

This study was approved by the American University of Beirut Institutional Review Board and is conformed to the guidelines set forth by the Declaration of Helsinki (Protocol Number: Bioch.GN.01). Patients with CHD seen at the Children's Heart Center of the American University of Beirut Medical Center (AUBMC) were sequentially recruited for the study. Informed consents/assents were obtained from all patients. Standard clinical evaluation included a complete physical exam, Electrocardiography (ECG), and Two-dimensional (2D) Trans-Thoracic Echocardiography (TTE) with color Doppler. Patients and available family members were interviewed for further documentation of disease history and pedigree information with phenotypes pointed out either by hearsay, prior to further documentation, or determined through medical records review. Patients with known syndromes were excluded from the study. For all patients and family members, blood or saliva was collected for DNA extraction. A total of 188 unrelated index CHD patients were recruited. Table 1 lists the phenotypes of the subjects recruited in the study. Once an *NKX2-5* variant was identified in an index patient, family members were recruited and screened for the variant. Selected patients and family members with confirmed *NKX2-5* mutations were asked to undergo additional studies (TTE, EKG, or Holter Monitor) for phenotyping, and in some cases medical records were reviewed for previous cardiac testing (e.g. coronary catheterization). Two patients (II-6 and III-1 in family B) also underwent cardiac electrophysiological studies. DNA of Lebanese control subjects (N = 80) was obtained from an existing registry of young individuals who self-reported as healthy and have no family history of CHD. The mutations identified were submitted to ClinVar database (Accession numbers SCV000188640, SCV000188641, SCV000188642, and SCV000188643).

### Genetic Analysis

DNA was extracted from blood or saliva. Index patients from 182 families, as well as 80 Lebanese control individuals, received targeted sequencing using a panel of 119 genes implicated in CHD including *NKX2-5* (Supplementary Table S1). Patients from the remainder 6 families received dideoxy sequencing for all exons of the *NKX2-5* gene. Targeted sequencing was done on Illumina GAIIx or HiSeq 2000 sequencers. Target capture was done using the Agilent SureSelect Target Enrichment System (www.agilent.com). Fastq files (50 bp paired-end) were aligned with Novoalign (www.novocraft.com). Bam files were processed with Picard (picard.sourceforge.net) and the Genome Analysis Toolkit (GATK), version 2.3–9. GATK Unified Genotyper was used to call variants. Variants were annotated with snpEff. Only *NKX2-5* variants that are covered >10X, that are of high quality scores, and that are rare (minor allele frequency <1% in both the analyzed dataset and the Exome Sequencing Project) were selected. Copy number variations (CNVs) were called from the targeted sequencing data on the 182 cases and 80 controls using the recently described XHMM methodology^22^. All *NKX2-5* variants were confirmed by dideoxy sequencing on the patient as well as all family members to determine segregation. Members of one large family with consanguinity (Family A) received genomewide SNP linkage analysis on the Illumina Human Omni Express chip, version 12v1_A. Multipoint logarithm of odds (LOD) scores were calculated with Linkmap/Fastlink, using a 5-marker sliding window approach, with each SNP's score reflecting contributions from its four nearest neighbors. Autosomal dominant inheritance was assumed, with a penetrance of 95% and a disease gene frequency of 0.001.

## Results

### Lebanese CHD Cohort Structure

The phenotypes of the index patients from the 188 families are summarized in Table 1. Twenty-five patients (13.3%) had ASD. Sixty-three (33.5%) of the 188 CHD patients and 10 (40%) of the 25 ASD subset reported consanguinity (parents are first or second degree cousins). Of the 25 ASD cases, 8 (32%) were familial as defined by having a sibling, parent, aunt/uncle, or cousin with CHD. These included 5 dominant pedigrees (affected parents) and 3 recessive parents (unaffected parents). Of the 188 families, 25 (13.3%) were familial.

### *NKX2-5* Variants in the Study

Table 2 lists all *NKX2-5* variants with MAF < 1%. The prevalence of these variants in the CHD cohort was 5.3% (10/188). The prevalence of *NKX2-5* variants in the ASD cohort is 24% (6/25) and 2% (1/80) in the control group (p = 0.0006). There were two truncating variants seen in the ASD cases (mutations causing a frameshift) and none in controls (p = 0.14). No CNVs were detected in the *NKX2-5* gene. No homozygous or compound heterozygous variants were detected.

### Familial ASD Cases

**Figure 1:**
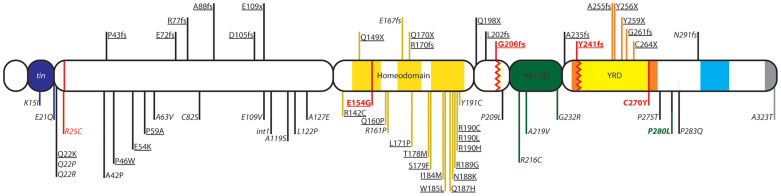
*NKX2-5* Mutations. The figure represents the different functional domains of the NKX2-5 protein with all previously published mutations overlaid. Single Nucleotide Variants (SNVs) are shown below the protein domains, and truncating insertions and deletions or nonsense mutations are shown above the protein. Bold and colored indicates variants reported in the current study, with green indicating variants in the control group and red indicating variants in CHD patients. Underlined variants refer to familial segregation, and italicized variants indicate a sporadic report of the variant in a case. Tin:Tinman Domain, YRD: tyrosine rich domain.

Three out of 5 (60%) familial dominant ASD kindreds had novel *NKX2-5* variants that segregated with the cardiac phenotype, two with truncating variants (p.G206fs and p.Y241fs) and one with a missense variant p.E154G (Figure 1). None of the three familial recessive ASD kindreds had *NKX2-5* variants.

**Figure 2:**
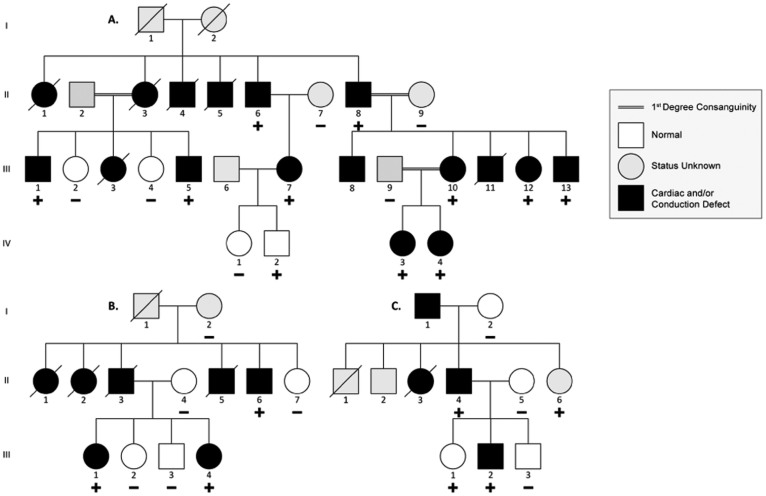
Pedigrees of Familial ASD Cases with Novel *NKX2-5* Mutations. Three families harbored novel *NKX2-5* mutations that segregated with the phenotype. Families A and B segregated novel deletions causing frameshift mutations, p.p.Y241fs and p.p.G206fs respectively. Family C segregated a novel missense variant p.E154G. + indicates heterozygous mutation − indicates no mutation.

Linkage analysis was performed on family A (Figure 2A and Sup. Table 2) prior to availability of the sequencing data. Two loci, *chr5: 166,000,000–177,000,000* and *chr11: 188,000,000–40,000,000* had equally high LOD scores of +2.0. The maximal theoretical LOD score of the family based on the samples analyzed was 2.1. The locus on Chr 5 contains the genes *NKX2-5, MSX2*, and *NSD1*. Targeted NGS on probands III:10 and III:11 of the family revealed 8 nucleotides deletion in exon 2 of *NKX2-5*; the deleted TACGGCGT nucleotides lead to a premature stop codon downstream of the Tyrosine residue at position 248 (p.p.Y241fs). The mutation was detected in all affected individuals, but was also detected in individual IV:2 who is 4 years old and with no ASD and no abnormal ECG reading (Figure 2A). In addition, one individual (II:8) had a concomitant missense variant (p.R25C) in *NKX2-5*. The calculated LOD score for p.Y241fs genotypes in Family A (penetrance = 95%) is +2.53.

In family B (Figure 2B, and Sup. Table 2), we found a single base deletion (G) in exon 2 in affected individuals III:1, III:4, and II:6 leading to a frame-shift mutation at the 206 Glycine residue (p.p.G206fs), and subsequently to a premature stop codon after 24 amino acids (residue 231). The mutation was not detected in 4 unaffected family members (Figure 2B). The calculated LOD score for p.G206fs genotypes in Family B (penetrance = 95%) is +1.44.

In Family C (Figure 2C, and Sup. Table 2), we detected a novel missense mutation (p.E154G) within the first helix of the homeodomain region (Figure 1). All affected members carry the mutation, while unaffected members have a normal genotype, with the exception of individual III-1 who carries the mutation and has a normal TTE and EKG at the age of 12 (Figure 2C). The calculated LOD score for p.E154G genotypes in Family C is −0.44 with penetrance of 95% and +0.25 with penetrance of 50%.

### Incompletely Penetrant *NKX2-5* Variants

A fourth family D (pedigree not shown) was found to have a novel missense variant (p.C270Y) in two affected siblings. Further screening of family members showed that the variant is inherited from an unaffected mother, and carried also by an unaffected uncle. A second uncle with CHD did not have the mutation. Therefore, it was determined that this variant alone does not fully explain disease in the family. Furthermore, the p.R25C variant was found in five CHD patients from families E, F, G, H, and I in addition to member II.8 from family A (Sup. Table 3,4). The p.R25C variant has been reported in many CHD sequencing cohorts with a burden in cases, and is believed to be incompletely penetrant^23^. In our cohort also, the transmission of the allele variant from phenotypically normal parents suggests that the *NKX2-5* variants p.C270Y and p.R25C alone are not-disease causing (Sup. Table 4). We hypothesized that variants in other cardiac genes that are, either *de novo* or inherited from one of the parents who does not carry the *NKX2-5* variant, cause the disease when occurring with it. To test this hypothesis, we interrogated our targeted sequencing data for rare (MAF < 1%) protein-changing variants in other candidate genes that are predicted to affect function by at least 2 out of 3 function prediction software for missense variants (PolyPhen II, PROVEAN, and SIFT). The list of the genes and the genotypes of all family members do suggest a combinatorial genetic interaction that would explain the observed phenotype in each case (Sup. Tables 3 and 4). In fact, there are concomitant *EVC2* and *CREBBP* missense variants in the index patient of family D with the *NKX2-5* p.C270Y variant. Among the patients carrying the *NKX2-5* p.R25C missense, patient F with ASD has a *de novo BMPR2* nonsense mutation, patient G with TOF has an in-frame deletion in *SOS1* and a missense in *NKX2-6* both inherited from the second parent, and patient I with PDA has a missense in *GATA4* inherited from the second parent (Sup. Table 4).

### Phenotype-Genotype Correlations

**Figure 3:**
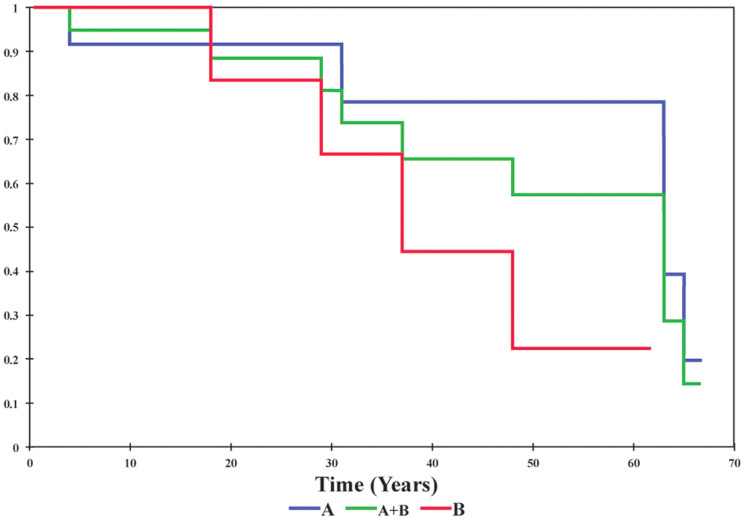
Kaplan-Meier Survival Estimate of Patients with NKX2-5 Truncating Mutations. Affected Patients from Families A, B, and combined are shown in blue, red and green respectively.

**Figure 4:**
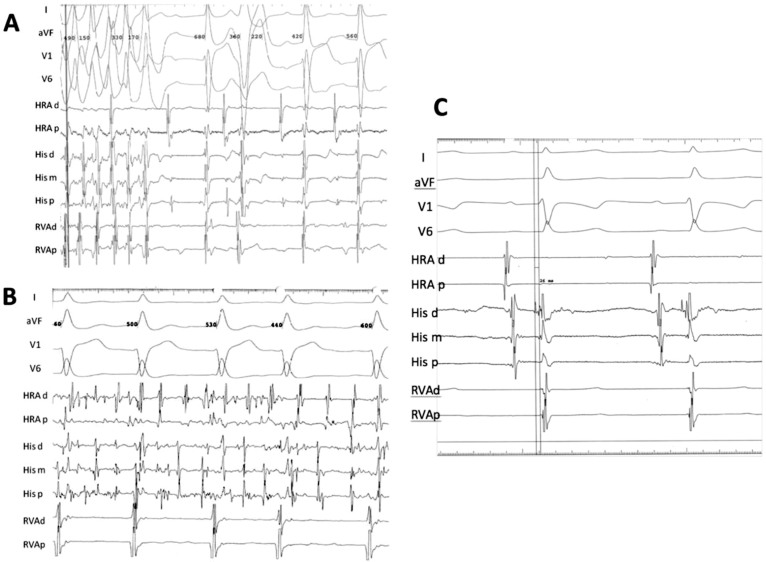
Electrophysiological Study Tracings of Two Patients with NKX2-5 Truncating Mutations. Multipolar electrode catheters were introduced percutaneously from the right femoral vein and positioned into the high right atrium (HRA) right ventricular apex (RVA) and His bundle region of two patients who carry NKx2.5 mutations. Bipolar electrograms (30 to 500 Hz) were displayed and stored using a digital recording system (EP MedSystems, West Berlin, NJ). One of the patients (B II:6) had no baseline ventriculo-atrial (VA) conduction, an atrial tachycardia that degenerated into atrial fibrillation requiring DC cardioversion (B), and spontaneous premature ventricular complexes isolated and in couplets with left bundle inferior axis morphology with early precordial transition (A). The other patient (B III:1) was in normal sinus rhythm, had a normal atrial-His (AH) interval, a short His-ventricular (HV) interval at 26 ms (C) [Normal HV interval: 35–55 ms] and no VA conduction was observed with or without isoproterenol infusion.

The phenotypes of familial *NKX2-5* mutations are reported in Sup. Table 2. Consistent with prior reports in the literature, ASD and Atrio-Ventricular Block (AVB) are the most common phenotype, with some patients manifesting other congenital malformations (TOF, VSD) and arrhythmias (atrial fibrillation). We report a large coronary sinus and coronary spasm in two patients, A III:1 and B II:6 respectively. Ten patients from the three families also had sudden cardiac death (SCD), nine of which are from families A and B carrying a truncating *NKX2-5* mutation. A Kaplan-Meier survival estimate shows a median age of SCD of 38 years in family B (p.G206fs) and 63 years in family A (p.Y241fs) (Figure 3). In order to further understand the occurrence of SCD in carriers of *NKX2-5* mutations, electrophysiological studies were performed on two patients with truncating *NKX2-5* mutations (B II:6 and B III:1), and revealed non-sustained ventricular tachycardia (NSVT) [Figure 4A] and an atrial tachycardia that degenerated into atrial fibrillation (Figure 4B) requiring synchronized electrical cardioversion in one patient, and a short His-ventricular (HV) interval at 26 ms suggesting pre-excitation in another patient (Figure 4C) [Normal HV interval: 35–55 ms]. One mutation carrier from family A (IV-2, age 4) and another from Family C (III:1, age 12) did not have any structural cardiac abnormality on TTE or rhythm abnormality on EKG.

## Discussion

### No founder *NKX2-5* Mutation in the Inbred Lebanese Population

It has been well-established that consanguinity is a risk factor for CHD, based on studies from consanguineous populations showing an increased occurrence of CHD primarily in first-cousin marriages^19^,^20^. In the current study, *NKX2-5* mutations occurred in 60% (3/5) of dominant familial ASD cases, but were absent from the three recessive ones suggesting that *NKX2-5* doesn't play a role in recessive forms of ASD even in consanguineous populations. Homozygous *NKX2-5* knockout mice are embryonic lethal, and it is thus, possible that occurrence of homozygous *NKX2-5* mutations is not detected in the study due to early embryonic lethality. In addition, recessive inheritance requires that heterozygous carriers can survive into fertility age, which was not possible prior to the development of surgical and interventional tools for CHD over the past two decades. That's why we could not identify recurrent variations in *NKX2-5* in the Lebanese population, except for the p.R25C variant, which is present in different populations at similar frequencies. In contrast, genetic investigation of other rare diseases in Lebanon by our group and others has identified founder mutations. In Familial Hypercholesterolemia (FH), we have shown that the “Lebanese allele” (p.C681X) at the LDL receptor (*LDLR*) gene causes 60% of the disease in Lebanon^24^. Studying L-Carnitine Deficiency (LCD), we have shown that a recurrent *SLC22A5* mutation (p.R254X) occurs in 3 out of 8 families (37.5%)^25^.

### The NKX2-5 C-terminal Domain is Indispensable for the Maturation of the Conduction System

The mutations in families A and B lead to truncation of the protein after the homeodomain region. Mutations cleaving the protein after the NK2-SD region as is the case in Family A, were found to reduce its ability to bind to DNA as dimers^15^, as well as reduce its transcriptional activity^18^. These findings can be attributed to the loss of a YRD conserved “pocket”^4^. The p.G206fs mutation in family B disrupts the protein prior to the NK2-SD domain. Truncating mutations occurring after the homeodomain region and prior to the NK2-SD domain have been shown to abolish binding ability and synergy with known cofactors *in vitro*^26^. The NK2-SD domain is thought to act as an intra-molecular repressor domain of C-terminal “activation domains”^27^. Interestingly, these mutations seem to loosen this auto-inhibitor control within *NKX*2-5 thus liberating its activation domain^28^. This gain of function should theoretically be considered as a counter balance for any possible defect if occurring in a homozygous manner, but since most of the mutations are affecting one allele, we hypothesize that inter and intra competitive squelching is occurring resulting in haploinsufficiency. Patients from family B with p.G206fs mutations have indeed similar phenotypes to patients with reported mutations deleting the NK2-SD domain^29^ and to family A patients with no deletions of this domain. The Kaplan-Meier survival curve shows a collective high burden of SCD. The survival estimate of patients with truncated NKX2-5 protein reaches 25% at age 30 and is around 75% at age 65. There is also a trend that supports a positive relationship between earlier sudden death and loss of the NK2-SD domain (Figure 1). EP study findings of NSVT and pre-excitation further support the conduction system disease in carriers of *NKX2-5* loss of function mutations (Figure 4).

The p.E154G missense mutation occurs at helix 1 of the homeodomain region of the protein responsible for DNA binding. All reported mutations occurring in this region present in patients with combined ASD and 1^st^ degree AV block. Functional studies of such proteins showed reduced DNA binding and cofactor interaction^15^,^16^,^17^,^18^. However, and in contrast to families A and B, there was only one SCD case in this family suggesting a crucial role for the C-terminal region of the NKX2-5 in conduction system disease.

We hypothesize that low levels of NKX2-5 proteins could impact the proper functioning of the postnatal heart in a similar way a cumulative physical stress affects both cardiac myocyte and conduction system cells. Pressure overload has been found in fact, to increase, and cause disorganization of, GJA5 and HCN4 expression within the peripheral conduction system while reducing lateral GJA1 coupling to myocardial cells^30^,^31^,^32^,^33^. This translates into an increase in transverse velocity of the cardiac impulse^33^ which might partially explain the early occurrence of AV block in the proband of family C (III2) with respect to a normal PR interval of his affected sibling with no ASD (III1); we hypothesize a delayed progression in this sibling to AV block as is the case with member II:6 of family B. Moreover, the absence of clear EKG findings in members with positive mutations (IV:2 of family A and III:2 of family C) does not rule out mild defects in the conduction system; factors such as increased heart rate alone are linked to disappearance of those EKG findings^34^.

Genome-wide association studies have linked *NKX2-5* variation to atrial fibrillation^35^. Three individuals with the mutation from families A and B are reported to have atrial fibrillation. Atrial vulnerability has been demonstrated in *NKX2-5* haploinsufficient mice; the atrial-like genetic program of pulmonary venous myocardium shifts to become pacemaker-like expressing HCN4 and down-regulating GJA5^36^. Moreover, the reduction in GJA5 expression alone has been linked to atrial fibrillation^37^ and increased spatial dispersion of refractoriness in the atria^38^.

On the other hand, disorganized^33^ and laterally distributed gap junctions within the ventricular conduction system increase the risk of ventricular arrhythmias^12^ possibly through re-entrant circuits^39^. EP results on member II:6 of family B reinforce those findings as they show an increased susceptibility to ventricular tachycardia with manipulation. This increased risk of ventricular arrhythmias could account for the increased burden of sudden death presenting within families A and B depicted in the Kaplan-Meier survival function plot (Figure 3).

### The NK Domain has a Role in Coronary Sinus Morphogenesis

Two individuals, III:1 of family A and II:6 of family B, in this study were found to have large coronary sinus during cardiac catheterization studies. The role of right-sided pressure overload in coronary sinus enlargement is well documented^40^. Interestingly, the presence of such findings in members with a formed septum (member II:6 of family B) alert to other pathologic processes. There are no previous reports on the association of any *NKX*2-5 mutations with such phenotype. Recent work, however, has identified a role for *NKX*2-5 in venous patterning of the heart in mice; *NKX*2-5-negative mesenchymal cell differentiation is essential for sinus horn development and patterning in an *NKX*2-5 rich heart^41^. These findings imply a more prominent role for *NKX2-5* in secundum atrial septal defect since the incorporation of the sinus horn in the lumen of the right atrium is associated with formation of a crescent-shaped septum secundum. Interestingly, previous documentation of other venous malformations have been reported in *NKX2-5* mutations^42^.

### The p.R25C Variant is not a Disease-Causing Variant

Missense mutations occurring outside the homeodomain region show evidence of non-penetrance^18^,^43^,^44^. Such a picture has led many to consider those mutations as benign polymorphisms^13^, while others postulated that pathogenicity is determined by other genetic interactions.

p.C270Y mutation in family D lies within the YRD region. The severity of the disease, and the absence of segregating phenotype within the family have prompted us to consider background genetic modifiers. Methodical filtering of “background” genetic mutations in the patient with single ventricle has identified a possible combination effect of three different mutations in *NKX2-5*, *EVC2* and *CREBBP*. The occurrence of mild phenotype in sibling III:1 with *NKX2-5* and *EVC2* mutations that is aggravated in the proband with an additional *CREBBP* mutation allows us to postulate a “multiple hit” phenomenon that is required within a single gene regulatory network and which is required to manifest in disease. Such a “multiple hit” phenomenon seems essential in disease manifestation considering molecular rescue mechanisms present that allow for disease buffering^45^,^46^.

In contrast, the missense p.R25C variant has already accumulated controversial views on its effect in congenital heart defects; it has been frequently detected in CHD patients, but it has also been reported in normal relatives and controls^43^,^44^,^47^,^48^, suggesting that it has little effect. In our study, we report the p.R25C variant in 6 CHD patients and one healthy control. Similarly, this mutation has been reported in 2/43 unaffected African Americans^48^, 2/185 healthy Turkish individuals^44^, and none out of 380 healthy Caucasians^23^. The p.R25C variant is also seen in the Exome Sequencing Project in 8 of 8536 European-American alleles (0.094%) and 115 of 4346 African-American alleles (2.65%), all as heterozygotes.

At the molecular level, the amino acid change at position 25 is thought to affect *NKX*2-5 homodimerization^15^ or reduce its post-translational activation^49^. Failure, however of the clinical features to segregate with the genotypes of affected families suggest that other mechanisms are at stake. First evidence of the non-functional role of p.R25C in CHD in our study, came from the concomitant double allele mutation of p.R25C and p.Y241fs in member II:8 of family A which did not exacerbate the phenotype of the affected individual. Second, in all the remaining 5 families (E–I), there are many members (Sup. Table 4) carrying the p.R25C variant and yet with no phenotype. In fact most of the 11 members from families E–I are healthy individuals based on thorough clinical assessment including EKG. These observations might suggest the need for other players that interact with *NKX*2-5 at the N-terminal domain in order to cause the phenotype linked to this particular mutation. Our approach to select the possible partners was based on the expression and function of genes of different families in the same cascade of cardiac morphogenesis. The associated genes (*GATA4*, *BMP4, CREBBP*, and *SOS1*) are all well known to interact or serve as downstream targets of *NKX2-5*^50^. However, these results are not conclusive and other genes could be involved.

### *NKX2-5* Variation Results in a Spectrum of Phenotypes

Considering all reported mutations in the gene, around 68% of those contribute to a phenotype of ASD^13^. However, out of the 35 familial cases reported to-date, including the three in the current study, 34 present with clinically documented ASD's and AV block (prevalence of 97.2%), and 12 with reported VSD's (34.3%) (Sup. Table 5). Contrary to prior understanding^34^,^51^,^52^, there does seem to be a genotype-phenotype correlation of *NKX2-5* mutations in CHD. All mutations causing truncation and all missense mutations of the homeodomain region lead to secundum ASD with AV block. Although ASD and AV block are the predominant manifestations, *NKX2-5* mutations result in a variety of phenotypes, structural and electrophysiological, often within the same family (Sup. Table 2). In explaining the incomplete penetrance, such as with individuals A IV-2 and C III-1 in this study, we hypothesize the presence of subclinical disease of the conduction system that might or might not manifest later in life, such as occurrence of SCD or Atrial Fibrillation at a young age.

## Conclusion

The finding of high burden of SCD in carriers of *NKX2-5* truncating variants highlights the need for genetic testing as well as close clinical follow up for best medical management in pateints suspected to have the disease. By combining our findings with a curated list of previously reported mutations, we propose to link the abnormal development of the conduction system to the homeodomain of the protein and its possible interaction with both the DNA and its partners. We also report novel phenotypes of large coronary sinus and coronary spasm detected on catheterization, and a high prevalence of SCD, which we further elucidated by EP studies.

## Additional Information

Note: Nucleotide sequence data reported are available in the ClinVar database under the accession numbers: SCV000188640, SCV000188641, SCV000188642, and SCV000188643.

## Supplementary Material

Supplementary Information Supplementary Dataset 1

## Figures and Tables

**Table 1:** Phenotypes of the Index CHD Cases in the Study

Phenotype	Number (percentage)
Atrial Septal Defect	25 (13.3)
Atrio-Ventricular Septal Defect	7 (3.7)
Coarctation	16 (8.5)
Pulmonary Atresia	9 (4.8)
Patent Ductus Arteriosus	6 (3.2)
Pulmonary Stenosis	15 (8)
Single Ventricle	22 (11.7)
Transposition of the Great Arteries	15 (8)
Truncus Arteriosus	3 (1.6)
Tetralogy Of Fallot	30 (16)
Ventricular Septal Defect	16 (8.5)
Aortic Stenosis/Bicuspid Aortic Valve	16 (8.5)
Other	8 (4.3)
Total	188

**Table 2:** List of *NKX2-5* Variants in the Study

*Nkx2-5* Variant	Locus and nucleotide change	ESP MAF (%)	PolyPhen Prediction	N (in 80 controls)	N (in 188 cases)	N (in 25 ASD cases)
p.R25C	5:172662014_G>A	0.94	Benign	1	6	2
p.E154G	5:172660086T>C	0	Possibly damaging	0	1	1
p.G206fs	5:172659929delC	0	NA	0	1	1
p.Y241fs	5:172659819-172659826delACGCCGTA	0	NA	0	1	1
p.C270Y	5:172659738C>T	0	Benign	0	1	1
