# Supplementary material for: NKX2-5 Mutations in an Inbred Consanguineous Population: Genetic and Phenotypic Diversity
Source: Sci Rep. 2015 Mar 6;5:8848. doi: 10.1038/srep08848 (PMC4351524; doi:10.1038/srep08848)
Supplement: Supplementary Information — Supplementary Dataset 1 [file srep08848-s1.docx]

**Supplemental Material for**

***NKX2-5* Mutations in an Inbred Consanguineous Population: Genetic and Phenotypic Diversity**

Running Title:

***NKX2-5* Mutations in an Inbred Consanguineous Population**

Ossama K. Abou Hassan^1,5#^, Akl C. Fahed^2,3,#^, Manal Batrawi^1^, Mariam Arabi^4^, Marwan M. Refaat^1,5^, Steven R. DePalma^2^, J. G. Seidman^2^, Christine E. Seidman^2, 6^, Fadi F. Bitar^1,4,*^, Georges M. Nemer^1,*^

Supplementary Table 1: List of Targeted Sequencing Genes

| ACTA2 | ELN | ISL1 | NOTCH1 | SESN1 |
| --- | --- | --- | --- | --- |
| ACTC1 | EP300 | JAG1 | NOTCH2 | SHOC2 |
| ACVR1 | ESCO2 | KCNJ2 | NPHP3 | SLC2A10 |
| AHSA2 | EVC | KIAA1841 | NSD1 | SOS1 |
| ANKRD1 | EVC2 | KIF3C | OSR1 | SRF |
| ASXL2 | FBN1 | KLF13 | PAPOLG | TBX1 |
| ATE1 | FBN2 | LBR | PCMTD2 | TBX20 |
| BCL11A | FGFR1 | LEFTY2 | PCSK5 | TBX3 |
| BCOR | FLNA | LRP2 | PEX1 | TBX5 |
| BMP4 | FOXC1 | MAX | PEX13 | TCF21 |
| BMP7 | FOXH1 | MED13L | PITX2 | TDGF1 |
| BMPR2 | FOXL2 | MEF2A | PKD2 | TFAP2B |
| C1orf106 | GATA4 | MEF2C | PLAGL1 | TLL1 |
| CCT4 | GATA5 | MGP | PPM1K | TWIST1 |
| CFC1 | GATA6 | MID1 | PPP3CA | UBR1 |
| CHD7 | GDF1 | MKKs | PQBP1 | USP34 |
| CITED2 | GJA1 | MSX1 | PROX1 | VEGFA |
| CREBBP | GJA9 | MSX2 | PTPN11 | VEGFC |
| CRELD1 | GPC3 | MYH11 | RAB10 | XPO1 |
| CRX | HAND1 | MYH6 | RAB23 | ZEB2 |
| CSRP1 | HAND2 | NF1 | RAI1 | ZFPM1 |
| CTNNA3 | HES1 | NFATC3 | RAI2 | ZFPM2 |
| DHCR7 | HES4 | NFATC4 | REL | ZIC3 |
| DNAI1 | HEY2 | NIPBL | ROR2 | ZNHIT3 |
| DQ983818 | HOXA1 | NKX2-5 | SALL1 |  |
| DVL1 | ID2 | NKX2-6 | SALL4 |  |
| EHMT1 | IGFBP4 | NODAL | SEMA3E |  |

Supplementary Table 2: Phenotypes of Members of Families A, B, and C

| Family  (NKX2-5 mutation) | Patient | Phenotype (age) |
| --- | --- | --- |
| Family A (p.Y241fs ) | I:1 | Unspecified arrhythmia (63) |
|  | II:1 | Septal Defect, Sudden Cardiac Death (65) |
|  | II:2 | Sudden Cardiac Death (31) |
|  | II:4 | Sudden Cardiac Death (63) |
|  | II:5 | Septal defect, Sudden Cardiac Death (63) |
|  | II:6 | Septal defect, AVB requiring PPM placement |
|  | II:8 | ASD, Afib |
|  | III:1 | Large Coronary Sinus, ASD II, Afib |
|  | III:3 | Septal defect, Sudden Cardiac Death (<1 year) |
|  | III:5 | Septal defect |
|  | III:7 | ASD |
|  | III:8 | ASD, VSD |
|  | III:10 | Septal defect |
|  | III:11 | Septal defect, Sudden Cardiac Death (4) |
|  | III:12 | ASD, First Degree AVB |
|  | III:13 | ASD, First Degree AVB |
|  | IV-2 | Normal TTE and EKG (4) |
|  | IV-3 | ASD |
|  | IV-4 | ASD |
| Family B (p.G206fs ) | I:1 | Septal Defect |
|  | II:1 | Septal Defect, Sudden Cardiac Death (18) |
|  | II:2 | Septal Defect, AVB requiring PPM placement |
|  | II:3 | Septal Defect, Sudden Cardiac Death (29) |
|  | II:5 | Septal Defect, Sudden Cardiac Death (37) |
|  | II:6 | Severe LAD and circ spasm, Large Coronary Sinus, Afib |
|  | III:1 | ASD, First Degree AVB |
|  | III:4 | ASD, First Degree AVB, RBBB |
| Family C (p.E154G) | I:1 | ASD |
|  | II:3 | Sudden Cardiac Death (<1 year) |
|  | II:4 | ASD, RBBB |
|  | II:6 | Unknown |
|  | III:1 | Normal TTE And EKG (12) |
|  | III:2 | ASD, First Degree AVB |

Afib: atrial fibrillation, AVB: atrioventricular block, LAD: Left anterior descending coronary artery, circ= circumflex coronary artery, RBBB: right bundle branch block, PPM: permanent pacemaker, VSD: ventricular septal defect, ASD: atrial septal defect, EKG: electrocardiogram, TTE: trans-thoracic echocardiography

Supplementary Table 3: Compound Heterozygous Variants in *NKX2-5* and Other Cardiac Genes in CHD Patients

|  | | | | | | | | PROVEAN PREDICTION | |
| --- | --- | --- | --- | --- | --- | --- | --- | --- | --- |
| Family | Phenotype | Gene | Amino Acid Variation | AC | Quality | Variation Type | SNP | SCORE | PREDICTION (cutoff=-2.5) |
| D | SV | CREBBP | p.A1869T | 5 | PASS | missense | . | -0.731 | Neutral |
| D | SV | EVC2 | p.L600P | 1 | PASS | missense | . | -3.384 | Deleterious |
| D | SV | NKX2-5 | p.C270Y | 1 | PASS | missense | . | -2.815 | Deleterious |
|  |  |  |  |  |  |  |  |  |  |
| E | VSD | BMP4 | p.H251Y | 1 | PASS | missense | . | -5.015 | Deleterious |
| E | VSD | NKX2-5 | p.R25C | 9 | PASS | missense | rs28936670 | -2.023 | Neutral |
|  |  |  |  |  |  |  |  |  |  |
| F | ASD | AHSA2 | p.R75K | 1 | PASS | missense | rs116242770 | -2.725 | Deleterious |
| F | ASD | BMPR2 | p.Q6* | 1 | PASS | nonsense | . | NA | NA |
| F | ASD | FMO5 | p.K166E | 6 | PASS | missense | rs58351438 | -2.546 | Deleterious |
| F | ASD | NKX2-5 | p.R25C | 9 | PASS | missense | rs28936670 | -2.023 | Neutral |
|  |  |  |  |  |  |  |  |  |  |
| G | TOF | NKX2-5 | p.R25C | 9 | PASS | missense | rs28936670 | -2.023 | Neutral |
| G | TOF | NKX2-6 | p.R217G | 1 | PASS | missense | . | -5.232 | Deleterious |
| G | TOF | SOS1 | Inframe |  |  |  |  |  |  |
| G | TOF | MYH6 | Duplication |  |  |  |  |  |  |
|  |  |  |  |  |  |  |  |  |  |
| H | DILV | NKX2-5 | p.R25C | 9 | PASS | missense | rs28936670 | -2.023 | Neutral |
|  |  |  |  |  |  |  |  |  |  |
| I | PDA | GATA4 | p.D425N | 3 | PASS | missense | rs56208331 | -2.827 | Deleterious |
| I | PDA | LRP2 | p.S4481L | 1 | PASS | missense | . | -3.506 | Deleterious |
| I | PDA | NKX2-5 | p.R25C | 10 | PASS | missense | rs28936670 | -2.023 | Neutral |

Supplementary Table 4: Genotype of the Families with Either the p.C270Y or p.R25C *NKX2-5*

| Family | Relation | Phenotype | Genotype | | | |
| --- | --- | --- | --- | --- | --- | --- |
| D | Father |  | Normal | *EVC2* L600P | | Normal |
| D | Mother |  | *NKX2-5* p.C270Y | Normal | | *CREBBP* p.A1869T |
| D | Patient | Single Ventricle | *NKX2-5* p.C270Y | *EVC2* p.L600P | | *CREBBP* p.A1869T |
| D | Sibling 1 | Aortic Valve thickening | *NKX2-5* p.C270Y | *EVC2* p.L600P | | Normal |
| D | Sibling 2 |  | Normal | Normal | | *CREBBP* p.A1869T |
| E | Father |  | Normal | *BMP4* p.H251Y | |  |
| E | Mother |  | *NKX2-5* p.R25C | Normal | |  |
| E | Patient | VSD | *NKX2-5* p.R25C | *BMP4* p.H251Y | |  |
| E | Sibling 1 |  | *NKX2-5* p.R25C | Normal | |  |
| E | Sibling 2 |  | *NKX2-5* p.R25C | Normal | |  |
| E | Sibling 3 | Small PFO | *NKX2-5* p.R25C | *BMP4* p.H251Y | |  |
| F | Father |  | *NKX2-5* p.R25C | Normal | |  |
| F | Mother |  | Normal | Normal | |  |
| F | Patient | ASD | *NKX2-5* p.R25C | *BMPR2* p.Q6* | |  |
| F | Sibling 1 |  | Normal | Normal | |  |
| F | Sibling 2 |  | *NKX2-5* p.R25C | Normal | |  |
| G | Father |  | *NKX2-5* p.R25C | Normal | | Normal |
| G | Mother |  | Normal | *SOS1* p.S1265in-frame | | *NKX2-6* p.R299G |
| G | Patient | TOF | *NKX2-5* p.R25C | *SOS1* p.S1265in-frame | | *NKX2-6* p.R299G |
| G | Sibling 1 |  | *NKX2-5* p.R25C | Normal | | *NKX2-6* p.R299G |
| G | Sibling 2 |  | *NKX2-5* p.R25C | Normal | | *NKX2-6* p.R299G |
| G | Sibling 3 |  | *NKX2-5* p.R25C | Normal | | *NKX2-6* p.R299G |
| H | Father | Normal echo | *NKX2-5* p.R25C |  | | |
| H | Mother |  | Normal |  | | |
| H | Patient | DILV | *NKX2-5* p.R25C |  | | |
| I | Mother |  | Normal | *GATA4* p.D425N | |  |
| I | Patient | PDA | *NKX2-5* p.R25C | *GATA4* p.D425N | |  |

PFO: patent foramen ovale, VSD: ventricular septal defect, ASD: atrial septal defect, TOF: tetralogy of fallot, DILV: double inlet left ventricular, *EVC2*: Elis Van Creveled 2, *BMP4*: Bone morphogenic protein 4, *CREBBP*: CREB binding protein, *SOS1*= Son of Sevenless 1, *SRF*: serum response factor, *GATA4*: GATA binding protein 4

Supplementary Table 5: Phenotype/Genotype of all reported *NKX2-5* Variants

| Domain | Chromosome Position, Amino Acid | Inheritance | Members with CHD Phenotype | Members genotyped | Phenotype | | | | | | | | |
| --- | --- | --- | --- | --- | --- | --- | --- | --- | --- | --- | --- | --- | --- |
|  |  |  |  |  | AV block | ASDII | VSD | TOF | Afib | Cardiac  death | | others | |
| tin | c.44A>T, p.K15I | s | 1 | 1 |  | 1 |  |  |  |  | |  | |
|  | c.61G>C, p.E21 |  | 1 | 3 |  |  |  | 1 |  |  | |  | |
|  | c.64C>A, p.Q22K | f | 5 | 5 | 5 | 5 |  |  |  |  | Pulmonary Stenosis | | |
|  | c.65A>C, p.Q22P | s | 1 | 1 |  |  |  | 1 |  |  |  | | |
|  | c.65A>G, p.Q22R | s | 1 | 1 |  | 1 |  |  |  |  |  | | |
|  | c.73C>T, p.R25C | s/f | >17 |  |  | Rep. | Rep. | Rep. |  |  |  | | |
|  | c.106C>A, p.R36S | f | 2 | 2 |  |  | 2 |  | 1 |  |  | | |
|  | c.124G>C, p.A42P |  | 1 | 2 |  |  |  |  |  |  | Ebstein | | |
|  | c.126_142delGCCCTCCTCCTGCATGC, p.P43fs | f | 4 | 3 | 2 | 4 |  |  |  |  | Mitral Valve fenestration | | |
|  | c.138C>G, p.P46W | f | 2 | 2 | 1 | 2 | 1 |  |  |  |  | | |
|  | c.160G>A, p.E54K | f | 3 | 3 |  |  |  | 3 |  |  |  | | |
|  | c.175>G, p.P59A | f | 5 | 3 | 2 | 1 | 5 |  |  |  |  | | |
|  | c.188C>T, p.A63V |  | 1 | 1 |  |  |  |  |  |  | L-TGA | | |
|  | c.215_221delAGCTGGG, p.E72fs | f | 6 | ?6-5 | 5 | 5 |  |  | 3 |  | Heterotaxia, Sinus Venosus | | |
|  | c.223_224delCG, p.R(75)77fs | f | 5 | 4 | 5 | 4 |  |  |  |  |  | | |
|  | c.244T>A, p.C82S | ? | 1 |  |  |  |  |  |  |  |  | | |
|  | c.262delG, p.A88fs | f | 4 | 4 | 4 | 3 |  |  |  |  |  | | |
|  | c.312delG, p.D105fs | f | 3 | 2 | 2 | 3 | 1 |  |  | 1 |  | | |
|  | c.325G>T, p.E109X | f | 5 |  | Rep. | Rep. | Rep. |  |  |  |  | | |
|  | c.326C>T, p.E109V |  | 1 | 1 |  |  | 1 |  |  |  |  | | |
|  | c.541T>C, p.L122P | f | 1 | 2 |  | 1 |  |  |  |  |  | | |
|  | c.380C>A, p.A127E |  | 1 | 1 |  | 1 |  |  |  |  |  | | |
| Homeodomain Site | c.424C>T, p.R142C | f | 17 | 13 | 11 | 10 | 3 | 1 |  |  | Pulmonary Stenosis, LV to RA connection, PDA | | |
|  | c.445C>T, p.Q149X | f | 6 | 6 | 5 | 4 | 3 |  |  |  |  | | |
|  | c.465T>C, p.E154G | f | 3 |  |  | Rep. |  |  |  |  | WPW | | |
|  | c.479A>C, p.Q160P | f | 4 |  | Rep. | Rep. |  |  |  |  |  | | |
|  | c.498_499insC, p.E167fs | s | 1 | 1 | 1 | 1 |  |  |  |  |  | | |
|  | c.508C>T, p.Q170X;  c.512insGC, p.R170fs | f | 6  3 | 4  3 | 3  2 | 4  2 |  |  |  | 2  1 | Left Ventricular Hypertrophy  Left Ventricular noncompaction | | |
|  | c.512T>C, p.L171P | f | 16 | 9 | 9 | 7 | 1 |  |  | 1 | Tricuspid Valve abnormalities | | |
|  | c.533C>T, p.T178M | f | 21  7  3 | 12  2  4 | 18  4 | 18  4  3 | 2 | 2 | 1  1 | 1  1 | Sub Valvular Aortic Stenosis  HLHS | | |
|  | c.536C>T, p.S179F | f | 4 | 4 | 2 | 4 |  |  |  |  | Pulmonary Stenosis | | |
|  | c.554G>T, p.W185L | f | 5 | 3 | 2 | 4 | 3 |  |  |  | Left Ventricular noncompaction, | | |
|  | c.561G>C, p.Q187H | f | 6  8 | 6  7 | 6  8 | 5  6 |  |  |  |  | Venous anomalies  Venous anomalies | | |
|  | c.564C>A, p.N188K | f | 5 | 5 | 5 | 5 |  |  |  | 1 | Tethered Tricuspid Valve, Ebsteins | | |
|  | c.565C>G, p.R189G | f | 5 | 5 | 5 | 4 |  |  | Rep. | 1 |  | | |
|  | c.568C>T, p.R190C;  c.569G>T, p.R190L;  c.569G>A, p.R190H | f | 3  2  3 | 1  2  3 | 1  2  3 | 2  2  3 | 2 |  |  |  | cribriform ASD  Tricuspid atresia | | |
|  | c.572A>G, p.Y191C |  | 1 | 1 | 1 | 1 | 1 |  |  |  |  | | |
|  | c.592C>T, p.Q198X | f | 6 | 3 | 3 | 6 |  |  |  | 3/6 | Mitral Valve fenestration | | |
|  | c.605_606delTG, p.L202fs | f | 8 | 3 | 7 | 7 | 2 |  |  |  |  | | |
|  | c.626C>T, p.P209L | s | 1 | 1 |  | 1 |  |  |  |  |  | | |
| NK2 | c.646C>T, p.R216C | s | 1 |  |  |  |  | 1 |  |  |  | | |
|  | c.656C>T, p.A219V |  | 1 | 2 |  |  |  | 1 |  |  |  | | |
|  | c.870G>A, p.G232R |  | 1 | 2 |  |  |  |  |  |  | Pulmonary Stenosis | | |
|  | c.701_702insTCCCT, p.A235fs | s | 1 | 2 | 1 | 1 |  |  |  |  |  | | |
|  | c.762delC, p.A255fs | f | 5 | 2 | 5 | 3 |  |  |  | 5/18 |  | | |
|  | c.768T>A, p.Y256X | f | 5 | 5 | 4 | 3 |  |  | 3 | 1/5 | unsustained V-Tachs | | |
|  | c.777C>A, p.Y259X | f | 7 | 7 | 7 | 6 | 3 |  |  |  | Double Outlet RV, Coarctation | | |
|  | c.792C>A, p.C264X | f | 6 | 1 | 6 | 4 |  |  |  |  |  | | |
|  | c.823C>A, p.P275T | s | 1 |  |  |  |  |  |  |  | Coarctation | | |
|  | c.839C>T, p.P280L |  | 1 | 1 |  |  |  |  |  |  |  | | |
|  | c.848C>A, p.P283Q |  | 1 | 1 |  |  | 1 |  |  |  | Patent Ductus, Aortic stenosis | | |
|  | c.871_873delAAC, p.N291del |  | 1 | 1 |  |  |  |  |  |  | Double Outlet RV | | |
|  | c.959delC, p.G293fs | f | 6 | 4 | 4 |  |  |  |  | 1 | Apical noncompaction | | |
|  | c.967G>A, p.A323T |  | 1 | 1 |  |  |  | 1 |  |  | |  | |
